# Association of a Family Integrated Care Model With Paternal Mental Health Outcomes During Neonatal Hospitalization

**DOI:** 10.1001/jamanetworkopen.2021.44720

**Published:** 2022-01-24

**Authors:** Nicole R. van Veenendaal, Sophie R. D. van der Schoor, Birit F. P. Broekman, Femke de Groof, Henriette van Laerhoven, Maartje E. N. van den Heuvel, Judith J. M. Rijnhart, J. Hans B. van Goudoever, Anne A. M. W. van Kempen

**Affiliations:** 1 Department of Pediatrics and Neonatology, OLVG, Amsterdam, the Netherlands; 2 Emma Children’s Hospital, Amsterdam University Medical Centres, University of Amsterdam, Vrije Universiteit, Amsterdam, the Netherlands; 3 Department of Psychiatry, OLVG, Amsterdam, the Netherlands; 4 Department of Psychiatry, Amsterdam University Medical Centres, Vrije Universiteit, Amsterdam, the Netherlands; 5 Department of Pediatrics and Neonatology, NoordWest ZiekenhuisGroep, Alkmaar, the Netherlands; 6 Department of Epidemiology and Data Science, Amsterdam Public Health Research Institute, Amsterdam University Medical Centres, Vrije Universiteit, Amsterdam, the Netherlands

## Abstract

**Importance:**

During newborn hospitalization in the neonatal unit, fathers often feel anxious and excluded from their child’s caregiving and decision-making. Few studies and interventions have focused on fathers’ mental health and their participation in neonatal care.

**Objective:**

To study the association of a family integrated care (FICare) model (in single family rooms with complete couplet-care for the mother-newborn dyad) vs standard neonatal care (SNC) in open bay units with separate maternity care with mental health outcomes in fathers at hospital discharge of their preterm newborn and to study whether parent participation was a mediator of the association of the FICare model on outcomes.

**Design, Setting, and Participants:**

This prospective, multicenter cohort study was conducted from May 2017 to January 2020 as part of the fAMily Integrated Care in the Neonatal Ward Study, at level-2 neonatal units in the Netherlands (1 using the FICare model and 2 control sites using SNC). Participants included fathers of preterm newborns admitted to participating units. Data analysis was performed from January to April 2021.

**Exposure:**

FICare model in single family rooms with complete couplet-care for the mother-newborn dyad during maternity and/or neonatal care.

**Main Outcomes and Measures:**

Paternal mental health was measured using the Parental Stress Scale: NICU, Hospital Anxiety and Depression Scale, Post-partum Bonding Questionnaire, Perceived (Maternal) Parenting Self-efficacy Scale, and satisfaction with care (EMpowerment of PArents in THe Intensive Care–Neonatology). Parent participation (CO-PARTNER tool) was assessed as a potential mediator of the association of the FICare model with outcomes with mediation analyses (prespecified).

**Results:**

Of 309 families included in the fAMily Integrated Care in the Neonatal Ward Study, 263 fathers (85%) agreed to participate; 126 fathers were enrolled in FICare and 137 were enrolled in SNC. In FICare, 89 fathers (71%; mean [SD] age, 35.1 [4.8] years) responded to questionnaires and were analyzed. In SNC, 93 fathers (68%; mean [SD] age, 36.4 [5.5] years) responded to questionnaires and were analyzed. Fathers in FICare experienced less stress (adjusted β, −10.02; 95% CI, −15.91 to −4.13; *P* = .001) and had higher participation scores (adjusted odds ratio, 3.424; 95% CI, 0.860 to 5.988; *P* = .009) compared with those in SNC. Participation mediated the beneficial association of the FICare model with fathers’ depressive symptoms (indirect effect, −0.051; 95% CI, −0.133 to −0.003) and bonding with their newborns (indirect effect, −0.082; 95% CI, −0.177 to −0.015).

**Conclusions and Relevance:**

These findings suggest that the FICare model is associated with decreased paternal stress at discharge and enables fathers to be present and participate more than SNC, thus improving paternal mental health. Supporting fathers to actively participate in all aspects of newborn care should be encouraged regardless of architectural design of the neonatal unit.

## Key Points

**Question:**

Is there an association between the neonatal care setting—a family integrated care (FICare) model in single family rooms with complete couplet-care for the mother-newborn dyad vs standard neonatal care in open bay units—and mental health and participation outcomes among fathers of preterm newborns?

**Findings:**

In this cohort study of 263 fathers, fathers in the FICare model perceived less stress and participated more in caring for their newborns compared with those in standard care. Participation mediated the beneficial association of the FICare model on fathers’ depressive symptoms and parent-newborn bonding.

**Meaning:**

These findings suggest that supporting fathers to actively participate in all aspects of care of preterm newborns should be encouraged regardless of the neonatal unit’s architectural design.

## Teaser

This cohort study examines the association of a family integrated care model vs standard neonatal care with mental health outcomes among fathers of preterm neonates.

## Introduction

Parents can experience hospitalization of their preterm newborn in the neonatal intensive care unit (NICU) as very stressful.^1,2^ Integrating the family as a relevant and irreplaceable part of the health care team and creating an environment welcoming continuous parental presence^3^ and active participation in neonatal care, or family integrated care (FICare), has been shown to be beneficial for mothers and their newborns.^4,5,6^

In addition to the mothers, fathers (or partners) also play an important role during newborn hospital stay and newborn development.^7^ In animal models, paternal presence early in life is associated with increased survival^8^ and improved social behaviors and emotional functions in offspring later in life.^9^ During the NICU stay of their newborn, human fathers often feel excluded from their newborn’s caregiving and decision-making.^2^ They are expected to support mothers and participate in care of their newborn, but they can also experience trauma, anxiety, and depression following preterm birth.^10,11,12^ They can struggle to combine a sustained presence in the NICU while maintaining employment and domestic responsibilities outside the NICU.^13^ Additionally, fathers can develop feelings of insecurity, helplessness, and a lack of control if they are not involved in their newborns’ care.^14^ Among mothers, FICare is associated with less stress,^6^ but it is unknown through which mechanisms. For fathers, little research has been conducted concerning their perinatal experiences in the event of prematurity and, specifically, studying the association of the neonatal care setting and father’s participation in newborn care with paternal mental health outcomes.

The primary objective was to study the association of the FICare model in single family rooms with complete couplet-care for the mother-newborn dyad vs standard neonatal care (SNC) in open bay units with mental health outcomes (stress, anxiety, depression, impaired father-newborn bonding, self-efficacy, and satisfaction) among fathers at discharge of their preterm newborn. The secondary objective was to study whether parent participation was a mediator of the association of the FICare model on paternal mental health.

## Methods

### Study Design

This study is part of the fAMily Integrated CAre in the Neonatal Ward Study (eAppendix 1 in the Supplement), a prospective, observational, cohort study comparing the FICare model with SNC in open bay units. The primary outcome is neurodevelopment in preterm newborns at the corrected age of 2 years.^15^ Mental health outcomes in parents are also studied in the short and long term. This study follows the Transparent Reporting of Evaluations With Nonrandomized Designs (TREND) reporting guideline for nonrandomized studies and A Guideline for Reporting Mediation Analyses of Randomized Trials and Observational Studies (AGReMA-SF).^16,17^ This study was approved by the medical ethical review committee of Medical Research Ethics Committees United Nieuwegein, the Netherlands.

**Figure 1.**
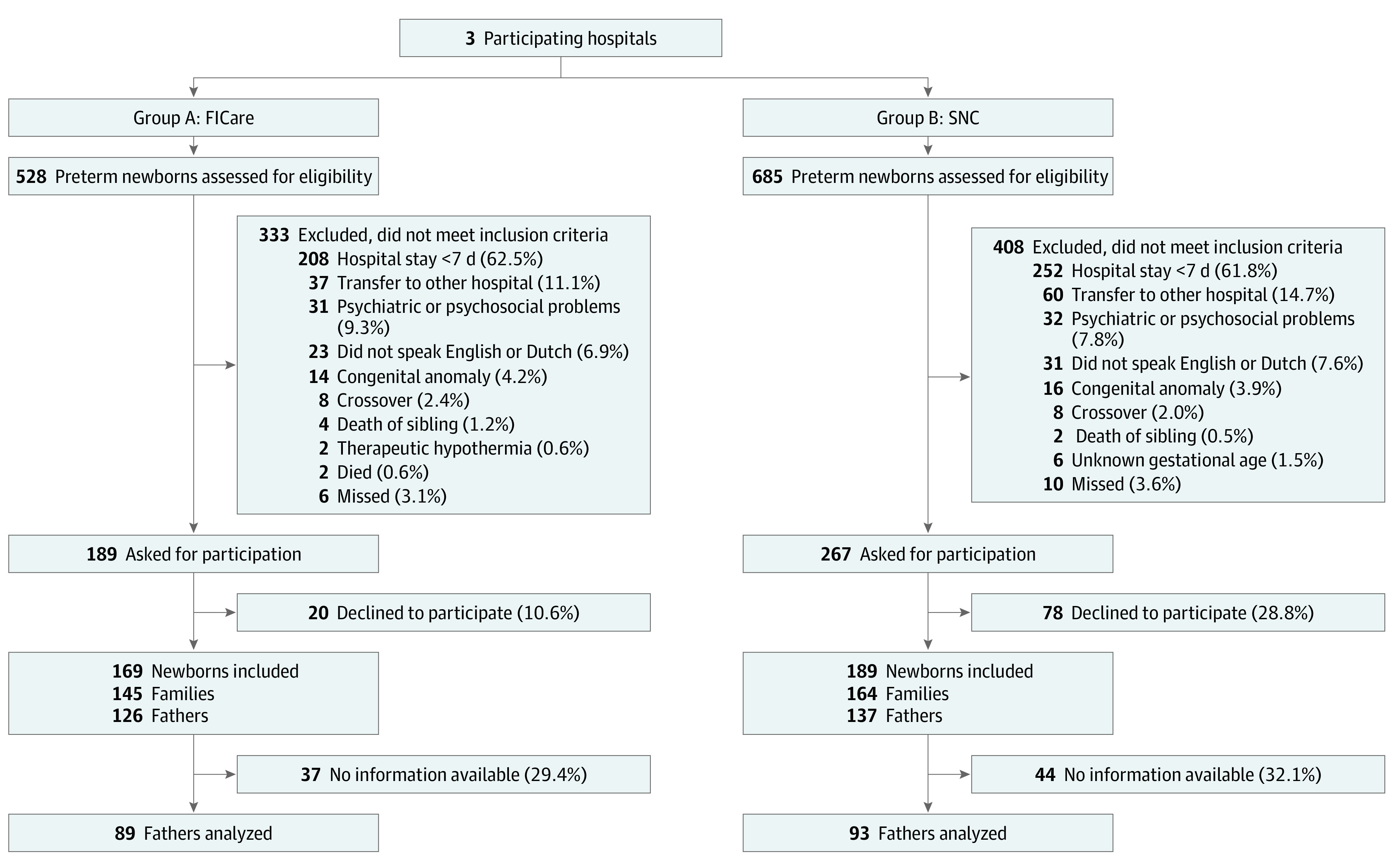
Flow Diagram FICare indicates family integrated care; SNC, standard neonatal care.

All newborns born in or transferred to level-2 neonatal wards participating in the study (1 exposure and 2 control sites) in the Netherlands were eligible. Preterm newborns (<37 weeks’ gestation) with a hospital stay longer than 7 days and their parents were included after the parents provided written informed consent. For this study, we analyzed the fathers of the families. We also included same-sex couples because we recognize and respect that there are people having children who may not identify as father or mother. For the sake of clarity, we use the term *fathers* for partners of the newborn’s mother who will assume a parental role. Exclusion criteria were severe psychosocial problems (parents with active psychiatric illness [ie, psychosis] and/or under supervision of child services), parents nonproficient in Dutch or English, newborn congenital abnormalities likely to influence neurodevelopment, or if death of an newborn occurred (see eAppendix 1 in the Supplement). Figure 1 shows the study enrollment flow chart.

### Exposure (FICare Model)

Within a large teaching hospital with a level-2 neonatal unit in Amsterdam, the Netherlands, an innovative FICare model was set up including complete mother-newborn couplet-care in single family rooms with rooming-in facilities with a concomitant participation program for parents and recurring education for staff. In this setting, integration between maternal and neonatal services was achieved for all newborns and their families^18^; mothers never had to be separated from their newborns during hospital stay when neonatal and/or maternity care was needed (couplet-care). Fathers could be continuously present with the family during obstetric, maternity, and neonatal care (eFigure 1, eFigure 2, and eFigure 3 in the Supplement). In addition, parents were trained to be the primary caregiver of their newborn, and nurses supported, instructed, and counseled parents.^19^ Parents were invited but not obligated to be present more than 8 hours per day, and rooming-in facilities were present.^20^

Parents were actively encouraged to participate in all aspects of their newborn’s care as much as they felt comfortable with, such as (but not limited to) providing feedings by nasogastric tube, breast, or bottle; providing skin-to-skin care; weighing; and regulating temperature control. Family-centered rounds were implemented and included active parental participation in shared decision-making on daily medical rounds and involvement in the process of patient management.^4,21^ In addition, parents received group education sessions to learn on all aspects concerning (preterm) newborn and family health.^4,22^

### Control Group (SNC)

Two different teaching hospitals with level-2 neonatal units in Amsterdam and Alkmaar, the Netherlands, were control centers in the study. Within these centers, maternity and neonatal care services were separated from each other. Ill or preterm newborns born at less than 35 weeks of gestation, weighing less than 2000 g, or in unstable condition were transferred to the neonatal unit. Maternity care was delivered in a ward separate from the neonatal ward. The neonatal units were set up with open bay units (eFigure 4 in the Supplement). Each incubator was separated by a curtain and had a chair available for parents. Nurses involved parents as much as possible in the care of their newborn. Parents could sign up for weekly updates with the pediatrician. Daily rounds were performed between the nursing staff and pediatrician, without the presence of the family. Nurses usually updated parents after decisions were made during daily rounds. No facilities were present for parents to room-in with their newborn during hospital stay.

### Data Collection

Included fathers were asked to complete mental health–related questionnaires at admission and discharge regarding stress (Parental Stress Scale: NICU [PSS-NICU]; maximum score, 130, with higher scores indicating more stress),^23^ anxiety and depression (Hospital Anxiety and Depression Scale; maximum score, 42, with higher scores indicating more depressive symptoms),^24^ parent self-efficacy (Perceived [Maternal] Parenting Self-efficacy Scale; maximum score, 80, with higher scores indicating more self-efficacy),^25^ and impaired parent-newborn bonding (Post-partum Bonding Questionnaire; maximum score, 125, with higher scores indicating more impaired parent-newborn bonding).^26^ Fathers also completed questionnaires regarding satisfaction with care at hospital discharge (EMpowerment of PArents in THe Intensive Care–Neonatology; maximum score, 6, with higher scores indicating more satisfaction)^27^ and how they participated and collaborated with health care staff in neonatal care using the CO-PARTNER^28^ tool (maximum score, 62, with higher scores indicating more participation and collaboration in neonatal care) (see eAppendix 1, eTable 1, and eTable 2 in the Supplement for an elaboration and sample size calculations). Finally, fathers completed a general questionnaire with details on their education, current job, and the cultural background with which they identified most (classified by the participant), smoking, alcohol, and recreational drug use. To improve response rates, fathers were reminded up to 2 times (7 and 14 days after initial questionnaires were sent).

### Statistical Analysis

We performed independent *t* tests for normally distributed data or Mann-Whitney *U* tests for nonnormally distributed data. χ^2^ tests were used to test for differences in binary outcomes. All tests were 2-sided. If expected cell counts were 5 or less, we calculated differences with the Fisher exact test.

Baseline characteristics between fathers with and without outcome variables at discharge were compared. We assumed that the data were missing-at-random. We used the proposed guidance as explained by Sterne et al^29^ for missing data and applied the multivariate imputation by chained equations (mice) procedure with parcel summary scores to missing data at the item level.^30^ All variables used in the analyses were included in the imputation model, as well as auxiliary variables related to the probability of missing data or to the variables with missing data itself. Variables that were multicollinear with other included variables were excluded from the imputation model. For all data sets, we performed 20 imputations and 50 iterations to obtain imputed data sets (see eAppendix 1 in the Supplement). Convergence was checked graphically with convergence plots. All analyses were performed on the imputed data sets, and results were pooled by using Rubins rules.^31^

To study associations between the FICare model and outcomes in fathers, we performed multivariable linear or logistic regression in imputed data sets. For nonnormally distributed outcome data, we first applied a (natural) logarithmic or square root transformation to obtain normal distribution, or if unsuccessful, dichotomized outcomes. Potential confounders and modifiers were identified from the literature and assessed using statistical analyses (see eAppendix 1 in the Supplement).

**Figure 2.**
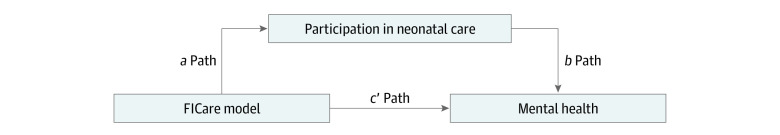
Parent Participation as a Mediator of the Association of the Family Integrated Care (FICare) Model on Mental Health Outcomes in Fathers

To study parent participation as a potential mediator of the observed association of the FICare model with mental health (ie, the *c*-path),^32^ we performed mediation analyses on the imputed data set.^20,32^ In addition to the total association model, 2 linear regression models were fitted. In single mediator models, total parent participation was included as individual potential mediator of different mental health outcomes in fathers (Figure 2). In the first regression model, the association of the FICare model with the mediator was estimated (*a*-path). In the second regression model, the association of the mediator with outcomes (*b-*path) and the direct effect size of the FICare model with outcomes (*c’*-path) were calculated. Crude and adjusted mediation analyses were performed. In the adjusted analyses, confounders were added to all models. We calculated the indirect effect size as the product of the *a* and *b* coefficients. We estimated bootstrap 95% CIs based on 1000 bootstrap resamples around the indirect effect sizes.^20,33^

We used R statistical software version 3.6.1 (R Project for Statistical Computing),^34^ including the mice package for multiple imputation^35^ and the boot package for the bootstrap 95% CIs.^36^ For all tests, *P* < .05 was considered significant. Data analysis was performed from January to April 2021.

## Results

A total of 309 families were included in this study, with 358 newborns and 559 parents (296 mothers and 263 fathers). One hundred twenty-six fathers consented to participate in the FICare model, and 137 fathers participated in SNC. Eighty-nine fathers (71%) in the FICare model (mean [SD] age, 35.1 [4.8] years; 82 male [98%]) and 93 fathers (68%) in the SNC model (mean [SD] age, 36.4 [5.5] years; 85 male [99%]) completed questionnaires and were analyzed (see eAppendix 2 in the Supplement). No differences were found in baseline characteristics between fathers who were responders and nonresponders (eTable 3 and eTable 4 in the Supplement). We included 3 same-sex partners, 2 in FICare and 1 in SNC. For baseline characteristics, see Table 1. An imbalance in the gestational ages was present between the 2 groups; newborns in the FICare model had lower gestational ages (median [IQR], 32 weeks 1 day [30 weeks 1 day to 35 weeks 0 days] vs 34 weeks 0 days [32 weeks 0 days to 35 weeks 0 days]; *P* = .008, Mann-Whitney *U* test) and longer hospital stays (median [IQR], 39 [15 to 58] days vs 21 [14 to 36] days; *P* < .001, Mann-Whitney *U* test) compared with the SNC group. Fathers in the FICare group experienced a higher level of stress at birth than fathers in the SNC care group (mean [SD] score, 3.2 [1.3] vs 2.7 [1.2]; *P* = .03, Mann-Whitney *U* test).

**Table 1.** Baseline Characteristics of Fathers

Characteristic	Participants, No./total No. (%)^a^		*P* value
	FICare group (n = 89)	SNC group (n = 93)	
Age, mean (SD), y	35.1 (4.8)	36.4 (5.5)	.11
Sex			
Female	2/84 (2)	1/86 (1)	.62^b^
Male	82/84 (98)	85/86 (99)	
University degree	74/82 (90)	75/85 (88)	.87
Paid job	71/82 (87)	72/84 (86)	.51
Work time, mean (SD), h/wk	39.7 (4.9)	40.9 (7.6)	.27
Identifies with Dutch cultural background	75/84 (89)	71/86 (83)	.30
Stress of pregnancy score, mean (SD)^c^	2.1 (1.3)	2.2 (1.4)	.53
Stress of birth score, mean (SD)^c^	3.2 (1.3)	2.7 (1.2)	.03
Gestational age, median (IQR) [range]	32 wk 1 d (30 wk 1 d to 35 wk 0 d) [24 wk 5 d to 36 wk 6 d]	34 wk 0 d (32 wk 0 d to 35 wk 0 d) [25 wk 3 d to 36 wk 6 d]	.008
Inborn newborn	40/89 (45)	61/93 (66)	.008
Singleton pregnancy	74/89 (83)	80/93 (86)	.74
First child upbringing	61/83 (73)	57/85 (67)	.46
Plan for upbringing together with partner	83/83 (100)	80/83 (96)	.25^b^
Smoking	8/78 (10)	12/82 (15)	.53
Use of drugs	4/78 (5)	2/80 (3)	.44^b^
Use of psychotropic drugs	0/87	1/92 (1)	.11^b^
Alcohol use	47/78 (60)	54/81 (67)	.50
Anxiety and depression score at admission, median (IQR)^d^	8 (3 to 14)	5 (3 to 7.8)	.32
Impaired parent-newborn bonding score at admission, median (IQR)^e^	9 (3 to 12.8)	9 (8 to 12)	.72
Parent self-efficacy score at admission, mean (SD)^f^	60.4 (6.9)	59 (5.9)	.43
Stress score at admission, mean (SD)^g^	43.2 (20.1)	41.5 (15.6)	.71

Abbreviations: FICare, family integrated care; SNC, standard neonatal care.

^a^ Denominators differ because of missing data.

^b^ Fisher exact test.

^c^ Maximum score is 5.

^d^ Measured with the Hospital Anxiety and Depression Scale; maximum score is 42, with higher scores indicating more depressive symptoms.

^e^ Measured with the Post-partum Bonding Questionnaire; maximum score is 125, with higher scores indicating more impaired parent-newborn bonding.

^f^ Measured with the Perceived (Maternal) Parenting Self-efficacy Scale; maximum score is 80, with higher scores indicating more self-efficacy.

^g^ Measured with the Parental Stress Scale: NICU; maximum score is 130, with higher scores indicating more stress.

At discharge, 156 of 182 fathers (86%) completed questionnaires regarding their mental health and participation in newborn care during hospital stay (eTable 5, eTable 6, and eTable 7 in the Supplement). At discharge, fathers’ total stress score in the FICare model was lower than those of fathers in SNC units (adjusted β, −10.02; 95% CI, −15.91 to −4.13; *P* = .001) (Table 2 and eTable 8 in the Supplement). Fathers experienced less stress due to the environment and newborn behaviors in the FICare model (adjusted β, –5.748; 95% CI, −10.140 to −1.356; *P* = .01) compared with SNC. They also experienced less stress due to changes in their parental role in the FICare model (adjusted β, −4.271; 95% CI, −6.536 to −2.006; *P* < .001).

**Table 2.** Fathers’ Participation in Neonatal Care During Hospital Stay and Mental Health Outcomes at Discharge^a^

Variable	Mean (SD)		β (95% CI)	*P* value	Adjusted β (95% CI)^b^	*P* value
	FICare (n = 89)	SNC (n = 93)				
Participation in neonatal care during hospital stay						
Presence, median (IQR), h/d	8.9 (2.3 to 15.5)	4 (2.1 to 5.9)	0.531 (0.268 to 0.794)^c^	<.001	0.582 (0.305 to 0.859)^c^	<.001
Presence >8 h per day, No. (%)	47 (52.8)	22 (23.7)	3.675 (1.793 to 7.531)^d^	<.001	4.942 (2.057 to 11.880)^d^	<.001
Total participation (maximum score 62)	45.9 (8.0)	43.6 (8.0)	4.405 (2.019 to 6.792)	<.001	3.424 (0.860 to 5.988)	.009
Domain 1, participation in daily care (maximum score 22)	16.2 (4.2)	15.5 (4.2)	1.369 (0.061 to 2.677)	.04	1.071 (−0.305 to 2.446)	.13
Domain 2, participation in medical care (maximum score 8)	5.1 (1.9)	4.5 (1.9)	1.192 (0.600 to 1.785)	<.001	0.861 (0.264 to 1.458)	.005
Domain 3, information gathering (maximum score 3)	2.3 (0.8)	2.4 (0.8)	−0.159 (−0.418 to 0.100)	.23	−0.274 (−0.541 to −0.008)	.04
Domain 4, advocacy and leadership (maximum score 3)	2.0 (1.0)	1.7 (1.1)	0.644 (0.308 to 0.980)	<.001	0.518 (0.162 to 0.874)	.005
Domain 5, time spent with newborn (maximum score 12)	7.9 (2.9)	7.2 (2.8)	1.280 (0.372 to 2.187)	.006	1.464 (0.463 to 2.464)	.005
Domain 6, comforting the newborn (maximum score 14)	12.2 (2.0)	12.2 (1.8)	0.114 (−0.459 to 0.687)	.69	−0.176 (−0.790 to 0.438)	.57
Mental health outcomes at discharge						
Stress Parental Stress Scale: NICU total score	40.8 (20.3)	49.4 (18.9)	−8.589 (−14.56 to −2.619)	.005	−10.02 (−15.91 to −4.130)	.001
Behavior and sights and sounds	29.7 (14.1)	34.8 (14.2)	−5.029 (−9.435 to −0.623)	.026	−5.748 (−10.14 to −1.356)	.011
Parental role alteration	11.1 (7.5)	14.6 (6.9)	−3.560 (−5.821 to −1.299)	.002	−4.271 (−6.536 to −2.006)	<.001
Depression and anxiety, median (IQR)	7.0 (3.6 to 10.4)	7.1 (3.3 to 10.9)	0.065 (−0.143 to 0.274)^c^	.54	0.023 (−0.183 to 0.230)^c^	.83
Self-efficacy score	63.8 (6.9)	62.2 (7.9)	1.648 (−0.790 to 4.086)	.18	1.459 (−1.100 to 4.018)	.26
Impaired parent-newborn bonding, median (IQR)	11.7 (5.1 to 18.1)	9.4 (4.4 to 14.4)	0.134 (−0.098 to 0.367)^c^	.26	0.137 (−0.109 to 0.382)^c^	.27
Satisfaction with care	5.2 (0.5)	5.2 (0.6)	0.055 (−0.111 to 0.220)	.52	0.085 (−0.085 to 0.255)	.32

Abbreviations: FICare, family integrated care; SNC, standard neonatal care.

^a^ Outcomes are from multiple imputed data sets.

^b^ Adjusted for gestational age, education, cultural background, age, stress at birth, work hours per week, upbringing plan, paternal smoking, and alcohol use.

^c^ Data are calculated after log transformation.

^d^ Data are odds ratio (95% CI).

### Participation During Hospital Stay

Fathers in the FICare model participated more in the care of their newborn compared with those in SNC (Table 2). Specifically, in the FICare model, fathers were more often able to be present and had higher total participation scores (adjusted odds ratio, 3.424; 95% CI, 0.860-5.988; *P* = .009). They searched less for information during hospital stay (CO-PARTNER tool domain 3) and participated more in medical care (domain 2, including tube feeding, monitoring of the newborn, regulation of visitation to newborn, and participating in daily rounds) than fathers in SNC. They also indicated being an advocate (domain 4) of their newborn more. No differences were found for comforting of the newborn.

### Mediation Analysis of Parent Participation on Outcomes

With mediation analyses, we could distinguish the direct effect of the FICare model (through the *c’* path) and indirect effect through increased parent participation (the *ab* path). Two different scenarios arose from mediation analyses (Table 3).

**Table 3.** Mediation Analysis of Parent Participation in Neonatal Care on Outcomes at Discharge^a^

Outcome	Association of the FICare model with mediator (participation), *a* pathway, mean (SE)	Associaton of mediator (participation) with outcome, *b* pathway, mean (SE)	Indirect effect (*ab* pathway), (95% CI)	Association of the FICare model with outcome, mean (SE)	
				*c*’- Pathway	*c*-Pathway
Crude analyses					
Self-efficacy	4.405 (1.207)	0.147 (0.074)	0.649 (−0.068 to 1.736)	0.997 (1.289)	1.648 (1.230)
Satisfaction with care	4.405 (1.207)	0.004 (0.006)	0.018 (−0.0312 to 0.082)	0.037 (0.088)	0.054 (0.084)
Depression and anxiety^b^	4.405 (1.207)	−0.016 (0.007)	−0.069 (−0.155 to −0.008)	0.134 (0.109)	0.065 (0.106)
Impaired parent-newborn bonding^b^	4.405 (1.207)	−0.024 (0.007)	−0.107 (−0.206 to −0.036)	0.242 (0.118)	0.134 (0.118)
Stress	4.405 (1.207)	0.255 (0.199)	1.121 (−0.610 3.282)	−9.715 (3.152)	−8.589 (3.023)
Adjusted analyses^c^					
Self-efficacy	3.424 (1.295)	0.133 (0.079)	0.457 (−0.119 to 1.357)	0.999 (1.341)	1.459 (1.292)
Satisfaction with care	3.424 (1.295)	0.005 (0.006)	0.018 (−0.022 to 0.075)	0.067 (0.088)	0.085 (0.086)
Depression and anxiety^b^	3.424 (1.295)	−0.015 (0.007)	−0.051 (−0.133 to −0.003)	0.074 (0.107)	0.023 (0.104)
Impaired parent-newborn bonding^b^	3.424 (1.295)	−0.024 (0.008)	−0.082 (−0.177 to −0.015)	0.219 (0.122)	0.137 (0.124)
Stress	3.424 (1.295)	0.223 (0.192)	0.763 (−0.627 to 2.517)	−10.78 (3.026)	−10.02 (2.977)

Abbreviation: FICare, family integrated care.

^a^ Outcomes are from multiple imputed data sets.

^b^ After log transformation, outcomes are from multiple imputed data sets.

^c^ Adjusted for gestational age, education, cultural background, age, stress at birth, work hours per week, upbringing plan, paternal smoking, and alcohol use.

#### Beneficial Outcomes Associated With the FICare Model That Were Explained by Parent Participation

Increased total participation in the FICare model was associated with fewer depressive symptoms (adjusted indirect effect, −0.051; 95% CI, −0.133 to −0.003) and lower impaired parent-newborn bonding scores (adjusted indirect effect, −0.082; 95% CI, −0.177 to −0.015) (*ab* path) (Table 3 and eTable 9 in the Supplement). No direct associations (*c’* path) for beneficial outcomes associated with the FICare model were observed for fathers’ depressive symptoms and parent-newborn bonding.

#### Beneficial Outcomes Associate With the FICare Model That Could Not Be Explained by Parent Participation

The FICare model was associated with less stress for fathers at discharge compared with fathers in SNC. Parent participation was not a mediator of this association (indirect effect, 0.763; 95% CI, −0.627 to 2.517). Fathers’ participation in neonatal care was not a mediator of the association of the FICare model for fathers’ self-efficacy at discharge (adjusted indirect effect, 0.457; 95% CI, −0.119 to 1.357) and also not for satisfaction with care (adjusted indirect effect, 0.018; 95% CI, −0.022 to 0.075).

## Discussion

In this cohort study in level-2 neonatal departments in the Netherlands, we found that fathers experienced benefits associated with implementing the FICare model in single family rooms with complete couplet-care for the mother-newborn dyad. In concordance with previous research,^3^ we found that in our FICare model NICU-related stress in fathers was considerably lower, and we add to the literature with possible explanations through mediation analyses. The reduced stress is in line with associations of FICare in mothers^4^ and single family rooms on mental well-being in fathers.^37^ Despite baseline differences in gestational age of the newborns, our results on mental health outcomes in fathers are in favor of the FICare model.

Our results suggest that it is especially the setting of the unit with single family rooms and complete couplet-care that supports fathers in reducing stress. Interestingly, the reduced stress level was not explained by increased participation in care.

Fathers have to provide emotional support to the mother,^38^ manage the family’s everyday life, and may have to return to work quickly^38^ during newborn hospitalization. They can perceive double burdens of concern for the well-being of the baby and the mother.^39^ Also, interpersonal factors, such as beliefs regarding fatherhood,^40^ health care professionals’ support,^41^ or parent-clinician communication,^42,43^ could potentially mediate the association between fathers’ participation in care and stress. In addition, education and support to fathers might need to be different than the support to mothers, but preferentially qualitative research is needed to explore this more in depth.

We found positive associations of the FICare model for fathers’ participation in care with depression and parent-newborn bonding. This finding complements previous literature^44^ by showing that the ameliorated mental health of fathers of preterm newborns is mediated through parent participation.^45,46^

The FICare model in this study is a multicomponent care model that addresses parent-newborn separation and promotes parent participation through different aspects, namely, the architectural design, integration of neonatal and maternity care, and a concomitant parent participation program. Solely addressing the architectural design does not improve mental health outcomes in parents and newborns.^3,47^ Also, it is possible to participate in care in standard care settings, even without additional FICare. We addressed these issues with mediation analysis, discerning the associations of different aspects of parent participation (assessed by the CO-PARTNER tool^28^) on fathers’ mental health outcomes. This is important for current NICU care settings that are unable to change to single family rooms or couplet-care, as stimulating and endorsing parent participation can also be augmented in neonatal units with open bay settings. Although we were unable to study the relationship of the newborn to the father in this study, we believe that increased interaction in care and improved father-newborn bonding will also lead to a stronger reciprocal (emotional) relationship over time between father and newborn, which will be beneficial to the newborn as well.^48^

Our results suggest that fathers in the FICare model experienced less stress compared with fathers in SNC. Future research could include measurement of biomarkers (eg, cortisol in hair or saliva) for better understanding of stress trajectories during newborn hospitalization and beyond.^49,50^ Equally, universal screening of all expecting fathers and families on vulnerability for mental health issues (eg, anxiety, depression, and risk for impaired bonding) should be performed antenatally as part of routine care.

### Strengths and Limitations

Strengths of this study include that we had a large sample of fathers. We included mediation analyses to identify and explain the hypothesized association of increased parent participation in the FICare model with outcomes in fathers, with advanced statistical techniques^20^ and a newly developed parent participation scale that was validated in fathers.^28^ Also, fathers had high consent and response rates.

This study also has limitations. Most of the scales we used in this study were validated in women and mothers, in the absence of suitable scales for fathers. Because fathers too can feel depressed or anxious and have trouble coping with the birth of an ill or preterm newborn,^51^ future research should focus on developing and validating scales for fathers specifically. This will enable us to compare interventions across studies, but also to further support fathers in real time and according to their specific needs.

In the absence of randomization due to hospital setting, we are unable to demonstrate causality between participation and outcomes. Our results might also suggest a bidirectional association between participation and outcomes. For instance, fathers who were highly stressed participated more or fathers who were less depressed participated more. Therefore, future studies should incorporate randomization for instance on the hospital level (ie, stepped-wedge cluster randomization) to evaluate hospital-based interventions.^52^ However, with remodeling toward single family rooms and the complexity of NICU care culture, this might be difficult.

## Conclusions

In this cohort study in level-2 neonatal units in the Netherlands, we found that an innovative FICare model with complete couplet-care for the mother-newborn dyad in single family rooms was associated with less perceived stress among fathers. In this FICare model, fathers can participate more, which is associated with fewer depressive symptoms and better parent-newborn bonding. Fathers should be enabled and supported to participate actively in all aspects of newborn care, and NICU care culture should be tailored to participation and the needs of fathers regardless of architectural design of the neonatal unit.

## Notes

Supplement. **eFigure 1.** Complete Couplet Care Setup **eFigure 2. **Single Family Room for Neonatal Level 2 Care **eFigure 3.** Family Participation in the Single Family Room **eFigure 4.** Standard Neonatal Care Setting With Open Bay Unit **eAppendix 1.** Supplemental Methods **eTable 1.** Scale Properties **eTable 2.** CO-PARTNER Tool **eAppendix 2.** Supplemental Results **eTable 3.** Response Rates of Fathers **eTable 4.** Baseline Characteristics of All Fathers Who Consented to Participate and Did or Did Not Fill Out Any Questionnaires During Infant Hospital Stay **eTable 5.** Baseline Characteristics of Included Fathers With or Without Filled Out Questionnaires at Discharge **eTable 6.** Missing Data in Baseline Characteristics of Fathers Included in the Analyses **eTable 7.** Baseline Characteristics Difference Between FICare Model and Two Standard Care Groups **eTable 8.** Answers on the PSS-NICU **eTable 9.** Associations Between Fathers’ Participation and Outcomes
eReferences
