# Supplementary material for: Association of a Family Integrated Care Model With Paternal Mental Health Outcomes During Neonatal Hospitalization
Source: JAMA Netw Open. 2022 Jan 24;5(1):e2144720. doi: 10.1001/jamanetworkopen.2021.44720 (PMC8787602; doi:10.1001/jamanetworkopen.2021.44720)
Supplement: Supplement. — eFigure 1. Complete Couplet Care Setup eFigure 2. Single Family Room for Neonatal Level 2 Care eFigure 3. Family Participation in the Single Family Room eFigure 4. Standard Neonatal Care Setting With Open Bay Unit eAppendix 1. Supplemental Methods eTable 1. Scale Properties eTable 2. CO-PARTNER Tool eAppendix 2. Supplemental Results eTable 3. Response Rates of Fathers eTable 4. Baseline Characteristics of All Fathers Who Consented to Participate and Did or Did Not Fill Out Any Questionnaires During Infant Hospital Stay eTable 5. Baseline Characteristics of Included Fathers With or Without Filled Out Questionnaires at Discharge eTable 6. Missing Data in Baseline Characteristics of Fathers Included in the Analyses eTable 7. Baseline Characteristics Difference Between FICare Model and Two Standard Care Groups eTable 8. Answers on the PSS-NICU eTable 9. Associations Between Fathers’ Participation and Outcomes eReferences [file jamanetwopen-e2144720-s001.pdf]

Supplemental Online Content

van Veenendaal NR, van der Schoor SRD, Broekman BFP, et al. Association of a family integrated care model with paternal mental health outcomes during neonatal hospitalization. *JAMA Netw Open*. 2022;5(1):e2144720. doi:10.1001/jamanetworkopen.2021.44720

eFigure 1. Complete Couplet Care Setup
eFigure 2. Single Family Room for Neonatal Level 2 Care
eFigure 3. Family Participation in the Single Family Room
eFigure 4. Standard Neonatal Care Setting With Open Bay Unit
eAppendix 1. Supplemental Methods
eTable 1. Scale Properties
eTable 2. CO-PARTNER Tool
eAppendix 2. Supplemental Results
eTable 3. Response Rates of Fathers
eTable 4. Baseline Characteristics of All Fathers Who Consented to Participate and Did or Did Not Fill Out Any Questionnaires During Infant Hospital Stay
eTable 5. Baseline Characteristics of Included Fathers With or Without Filled Out Questionnaires at Discharge
eTable 6. Missing Data in Baseline Characteristics of Fathers Included in the Analyses
eTable 7. Baseline Characteristics Difference Between FICare Model and Two Standard Care Groups
eTable 8. Answers on the PSS-NICU
eTable 9. Associations Between Fathers' Participation and Outcomes
eReferences

This supplemental material has been provided by the authors to give readers additional information about their work.

© 2022 van Veenendaal NR et al. *JAMA Network Open.*

eFigure 1. Complete Couplet Care Setup

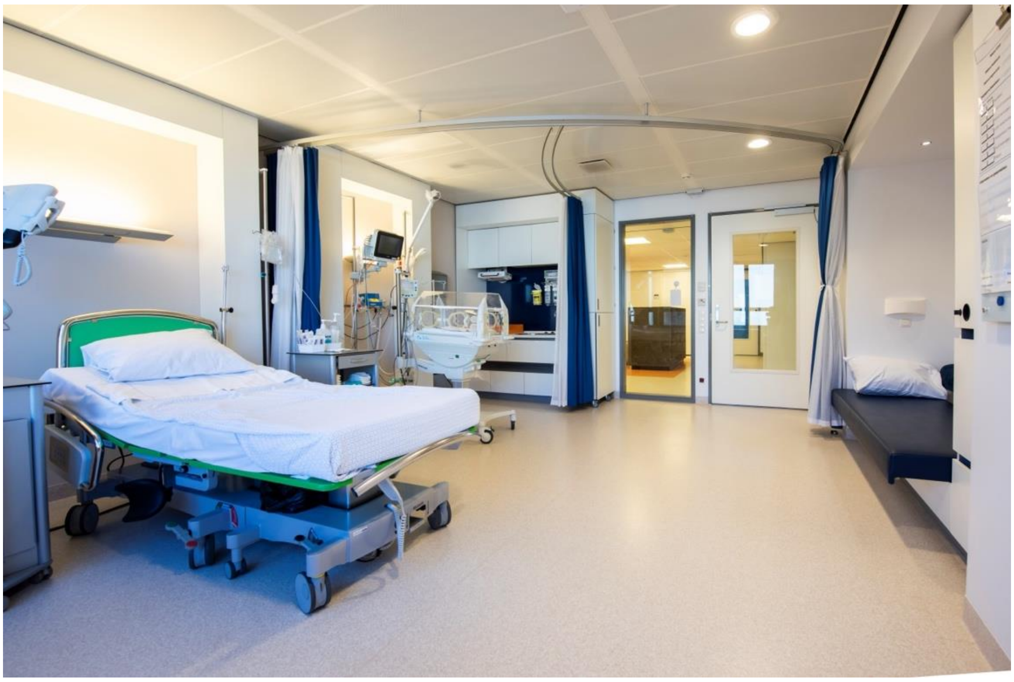

A Single-Family Room for highly complex maternity and neonatal Level 2 Care. Women and their newborns will remain in this suite for as long as both require specialized care, or at least for 7 days if the newborn requires specialized care. Fathers, too, can be present continuously. If after 7 days and one of them no longer needs specialized care, the woman and the newborn are transferred to a smaller single-family room, a room for highly complex maternity care and neonatal level 1 care or a room for neonatal level 2 care. All single-family rooms provide rooming-in facilities for one parent/partner. Printed with the permission of the audiovisual department OLVG Hospital, Amsterdam, the Netherlands, June 2020.

eFigure 2. Single Family Room for Neonatal Level 2 Care

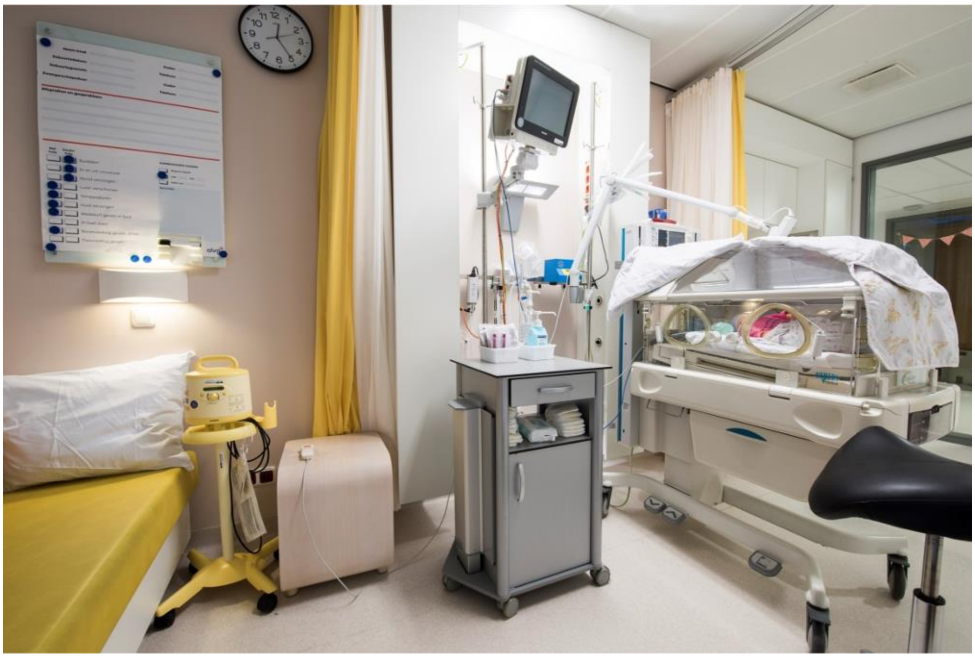

© 2022 van Veenendaal NR et al. *JAMA Network Open.*

eFigure 3. Family Participation in the Single Family Room

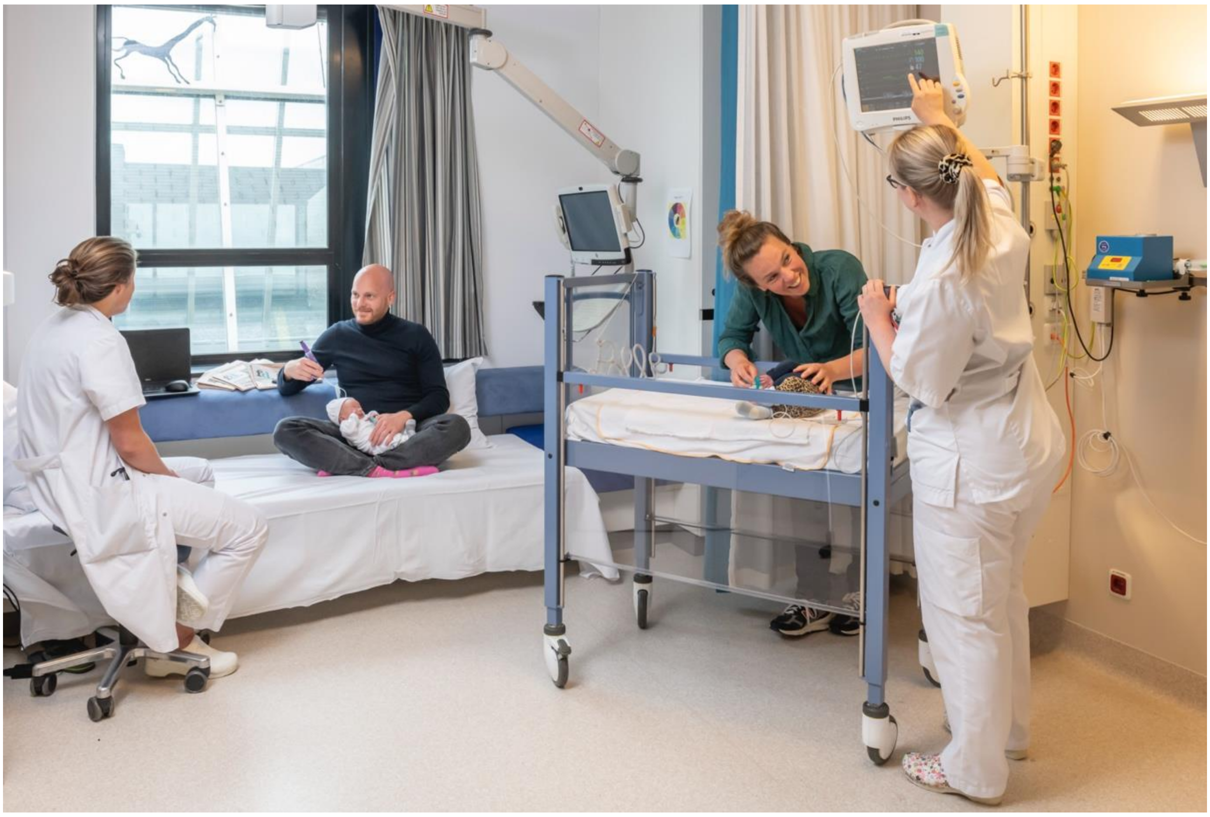

Depicted is a family with twin infants born at a gestational age of 32 weeks, together with a doctor and nurse specialized in neonatal care. The family stays continuously together in a single family room in our integrated neonatal-maternity ward. This enables both parents to participate, as equal partners in the medical team, in the care and medical decision making for their infants during hospital stay.
Printed with the permission of the audiovisual department OLVG Hospital, Amsterdam, the Netherlands, June 2020.

© 2022 van Veenendaal NR et al. *JAMA Network Open.*

eFigure 4. Standard Neonatal Care Setting With Open Bay Unit

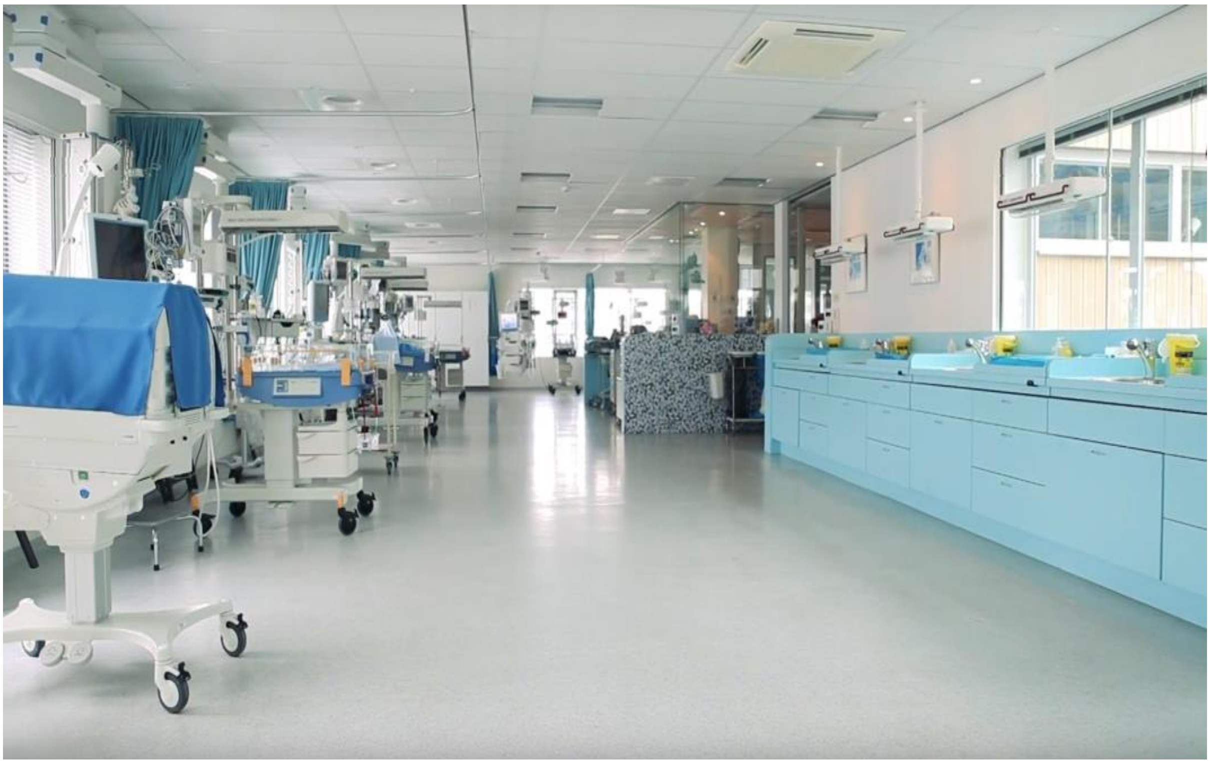

© 2022 van Veenendaal NR et al. *JAMA Network Open.*

eAppendix 1. Supplemental Methods
Scales Used in the Study

To measure stress, the Parental Stress Scale: Neonatal Intensive Care Unit (PSS-NICU) was used. This scale was developed by Miles in 1993 and measures the parental perception of stressors due to the physical, psychosocial and emotional environment of the neonatal intensive care unit[1]. This scale consists of 26 questions and was filled in both at admission and at discharge.. The PSS-NICU scale has 3 subdomains and measures the degree of stress experienced by parents during hospitalization related to alterations in their parental role, the appearance and behavior of their infant, and sights and sounds of the unit. Parents rate their experiences on a 5-point rating scale ranging from "not at all stressful" to "extremely stressful." In an update of the tool in 2007, sights and sounds of the environment (5 items) were combined with infant's appearance subscale (14 items) and scored as one subscale and Parental Role Alteration as the second subscale (7 items).[2]
If fathers did not experience the stressor, we transformed the score to "0".[3]
The Hospital Anxiety and Depression Scale was developed in 1983 and is used to measure the possible presence of anxiety and depressive complaints (Zigmond & Snaith, 1983). This scale has also been converted to Dutch, so that it is also available in this language (Spinhoven et al, 1997). Parents were asked to fill in this 14-item self-report at admission and at discharge.

Parent- and infant bonding
The Postpartum Bonding Questionnaire (PBQ), was devised by Brockington et al. (2001) as a screening instrument to detect bonding problems in obstetric and primary care services [4,5]. The PBQ is a 25- item scale reflecting a mother's feelings or attitudes towards her baby (e.g. ''I feel close to my baby'', ''My baby irritates me''). Participants rate how often they agree with these statements on a 6-point Likert scale ranging from always (score=0) to never (score=5) with low scores denoting good bonding. The PBQ has four subscales which reflect impaired bonding (Scale 1) (12 items, ranging from 0 to 60), rejection and anger (Scale 2) (7 items, scores ranging from 0 to 35), anxiety about care (Scale 3) (4 items, scores ranging from 0 to 20) and risk of abuse (Scale 4) (2 items, scores ranging from 0 to 10). Scale 1 (impaired bonding) has a sensitivity of 0.93 and a specificity of 0.85 in detecting mothers with a bonding disorder.

Parental empowerment and satisfaction
Parent satisfaction was measured using the EMpowerment of PArents in THe Intensive Care - Neonatology questionnaire[6]. This scale was developed and tested in a single center in the Netherlands, and available in Dutch. The domains covered are: Information (14 statements); Care and Treatment (20 statements); Parental Participation (nine statements); Organization (11 statements); and Professional Attitude (13 statements). The 57 statements divided in five domains provide a conceptualization of parent satisfaction within the neonatal ward from a family-centred care perspective[6].

Parental Self Efficacy
The Perceived (Maternal) Parenting Self-Efficacy (PMP-SE) tool, was used to measure perceived parental self-confidence when caring for the infant admitted to the Neonatal Ward [7]. The internal consistency reliability of the Perceived Maternal Parenting Self-Efficacy tool is 0.91, external/test-retest reliability is 0.96. A total of four conceptually unique subscales of parenting are: "Care taking procedures" (parents' perceptions of their ability to perform the activities and tasks related to the baby's basic needs like feeding). "Evoking behaviour(s)" (perceptions in their ability to elicit a change in the baby's behaviour, for example, soothing the baby). "Reading behaviour(s) or signalling" (perceptions in their ability to understand and identify changes in their baby's behaviour, for example, 'I can tell when my baby is sick'). "Situational beliefs"(parents' beliefs about their ability to judge their overall interaction with the baby). Responses to each item were recorded on a four point Likert scale ranging from 'strongly disagree' (score 1) to 'strongly agree' (score 4). A low score on this scale indicates a low parental self-efficacy.

© 2022 van Veenendaal NR et al. *JAMA Network Open.*

We previously validated a measurement tool on parent participation in neonatal care (the CO-PARTNER tool), which includes 6 domains: 1) daily care 2) medical care 3) information gathering 4) advocacy and leadership 5) time spent with infant 6) closeness and comforting the infant.[8] Total scores per domain were obtained by summing scores. For Domain 1, 2 and 6 we calculated 0 for 'the nurse does this', 1 for 'the nurse and I do this together' and 2 for 'I do this independently' (minimum scores 0 to 22, 8 and 14 respectively). For domain 3 and 4 'yes' was scored as 1, and 'no' as 0 (minimum scores 0 to 3). Non-applicable items were transformed to 0 (no participation in this item). For the domain Time Spent with Infant (3 items) quartiles were calculated resulting in 0 to 4 score (total score in domain 5 minimum 0 maximum 12). A total participation score was obtained by summing all domain scores. Minimum total scores were 0 and maximum 62. Additionally, we aimed to analyze in multiple mediation models the extent to which domain of participation contributes to ameliorated outcomes in the FICare model.

Inclusion and exclusion criteria of the AMICA study.

In order to be eligible to participate in this study, a subject had to meet all of the following criteria:

- Born between 24 and 36 6/7 weeks gestational age, with a postconceptional age ≤ 44 weeks on admission
- At least 1 week of hospital stay on the Neonatal Ward. Infants <35 weeks gestational age were recruited in the first days after birth, as their hospital stay is usually more than 7 days. Infants with a gestational age between 35 and 37 weeks were recruited after consultation with the attending physician, and were recruited when a hospital stay of >7 days was expected.
- Would visit the Outpatient Clinic of the OLVG East, OLVG West or NWZA after discharge or consents to gather required information through infant health centers
- Written informed consent from the parents

A potential subject who met any of the following criteria was excluded from participation in this study:

- Metabolic or chromosomal/syndromal diseases
- Therapeutic hypothermia for perinatal asphyxia
- Severe psychiatric or psychosocial problems i.e. parents under supervision of youth care
- Transfer to another hospital before discharge
- Parents unable to answer the questionnaires in Dutch/English
- Death of an infant

Sample size calculation

The primary outcome of the AMICA study is neurodevelopment in infants at 2 years of age. We pre-stratified the study population towards infants born <32 weeks of gestation with a previous admission to a level 3 NICU and infants that were born >32 weeks of gestation. Within each gestational age group, we did a power calculation for the primary outcome of neurodevelopment. We calculated to have 64 experimental subjects and 128 control subjects with power 0.90 (1-β) at a significance level of 0.05 (α) with a true difference in the outcome of neurodevelopment at the age of 2 years of ½ SD. To allow for 30% withdrawal we aimed to include 91 patients in group A and 182 patients in group B per risk group (post-

© 2022 van Veenendaal NR et al. *JAMA Network Open.*

intensive care versus inborn infants). A total of 546 infants who were hospitalised and their parents were expected to be included in this study.

For this study we calculated the power for the outcome on stress in fathers *post-hoc*.[11] The group sample sizes of 89 and 93 achieved 84% power to detect a difference of -8.6 between the null hypothesis that both group means are 40.8 and the alternative hypothesis that the mean of group 2 is 49.4 with known group standard deviations of 20.3 and 18.9 and with a significance level (alpha) of 0.05 using a two-sided two-sample t-test.

Variables used in multiple imputation model

For the imputation model we used the following strategy[12]

1. Include all variables that are part of the analysis model, including the dependent (outcome) variable.

2. Include additional (auxiliary) variables that are related to nonresponse

3. Include additional variables that are related to variables with missing values.

4. Exclude variables with a high correlation (> 0.90) that cause multi-collinearity problems.

5. Exclude variables with high percentages of missing data (> 50%).

The following variables were used in the multiple imputation model:

- Gestational age in days
- Inborn
- Work hours per week
- FICare model
- Singleton status
- Work hours per week
- Identifies with Dutch cultural background
- University degree
- HADS at admission/discharge*
- PBQ at admission/discharge*
- PMP at admission/discharge*
- PSS-NICU at admission/discharge*
- Participation at admission/discharge*
- Smoking
- Use of recreational drugs
- Alcohol use

Collinear variables

- Gestational age < 32 weeks
- Length of stay in NICU (with total gestational age)
- Length of stay total (with total gestational age)
- Paid work (with work hours per week)
- GA admission (with total gestational age)

© 2022 van Veenendaal NR et al. *JAMA Network Open.*

- Use of psychotropic drugs (n (%))

*depending on parcel mean summary score and which database was imputed.

Confounders and effect modifiers

Potential confounders and effect modifiers were identified from the literature and assessed using statistical analyses. We considered socio-economic class (including education and employment status), family composition (single parent vs co-parenting), older/other infants in the family, stress at birth, gestational age of infant, singleton status and mode of delivery. If the beta-regression coefficient differed at least 10% in regression analyses, this was used as an indication of statistical confounding, and the variable was included in the adjusted model. If collinearity was present, the strongest confounder (largest change in crude beta-coefficient) was used to adjust for.

© 2022 van Veenendaal NR et al. *JAMA Network Open.*

eTable 1. Scale Properties

	Scales	Minimum score	Maximum score	Internal consistency/ Cronbach's alpha (reference)
Stress	PSS-NICU	0	130	0·89-0·94 [1,2]
	Sights and sounds, behaviour of the infant	0	95	0.92 [2]
	Parental role alteration	0	35	0.92 [2]
Anxiety and depression	HADS	0	42	0.71-0.90 [9]
Self-efficacy	PMP-SE	20	80	0.91 [7]
Mother infant bonding	PBQ	0	125	0.87-0.78 [10]
Satisfaction with care (median scores over all items)	EMPATHIC-N	1	6	082 -0.95 [6]
Collaboration and participation in neonatal care	CO-PARTNER	0	62	NA [8]
	**Domain 1** *Participation in daily care*	0	22	0.934 [8]
	**Domain 2** *Participation in medical care*	0	8	0.558 [8]
	**Domain 3** *Information gathering*	0	3	0.745 [8]
	**Domain 4** *Advocacy and leadership*	0	3	0.855 [8]
	**Domain 5** *Time spent with infant*	0	12	0.839 [8]
	**Domain 6** *Comforting the child*	0	14	0.871 [8]

EMATHICN-N: EMpowerment of PArents in THe Intensive Care- Neonatology, HADS: hospital anxiety and depression score, PBQ: postpartum bonding questionnaire, PMP-SE: *The Perceived (Maternal) Parenting* Self-Efficacy, PSS-NICU: parental stress scale – neonatal intensive care unit

© 2022 van Veenendaal NR et al. *JAMA Network Open.*

eTable 2: CO-PARTNER Tool

Activity	Response
**Domain 1. Daily Care**	
1.Bath my child/clean my child with a washcloth.	○ The nurse does this<br>○ I do this together with the nurse<br>○ I do this independently (without the help of the nurse)<br>○ This is not applicable
2. Change my child's diaper.	○ The nurse does this<br>○ I do this together with the nurse<br>○ I do this independently (without the help of the nurse)<br>○ This is not applicable
3. Feed my child (breast or bottle).	○ The nurse does this<br>○ I do this together with the nurse<br>○ I do this independently (without the help of the nurse)<br>○ This is not applicable
4. Change my child's clothing.	○ The nurse does this<br>○ I do this together with the nurse<br>○ I do this independently (without the help of the nurse)<br>○ This is not applicable
5. Get my child out of the incubator/cradle.	○ The nurse does this<br>○ I do this together with the nurse<br>○ I do this independently (without the help of the nurse)<br>○ This is not applicable
6. Give my child medication.	○ The nurse does this<br>○ I do this together with the nurse<br>○ I do this independently (without the help of the nurse)<br>○ This is not applicable
7. Weigh my child.	○ The nurse does this<br>○ I do this together with the nurse<br>○ I do this independently (without the help of the nurse)

© 2022 van Veenendaal NR et al. *JAMA Network Open.*

	○ This is not applicable
8. Keep track of output (urination and defecation) of my child	○ The nurse does this<br>○ I do this together with the nurse<br>○ I do this independently (without the help of the nurse)<br>○ This is not applicable
9. Measure the temperature of my child.	○ The nurse does this<br>○ I do this together with the nurse<br>○ I do this independently (without the help of the nurse)<br>○ This is not applicable
10. Keep track of my child's weight.	○ The nurse does this<br>○ I do this together with the nurse<br>○ I do this independently (without the help of the nurse)<br>○ This is not applicable
11. Keep track of drinking and my child's feeds.	○ The nurse does this<br>○ I do this together with the nurse<br>○ I do this independently (without the help of the nurse)<br>○ This is not applicable
**Domain 2. Medical Care**	
12. Give tube feeding to my child.	○ The nurse does this<br>○ I do this together with the nurse<br>○ I do this independently (without the help of the nurse)<br>○ This is not applicable
13. Look at my child's monitor and handling accordingly (e.g. stimulating during a bradycardia).	○ The nurse does this<br>○ I do this together with the nurse<br>○ I do this independently (without the help of the nurse)<br>○ This is not applicable

© 2022 van Veenendaal NR et al. *JAMA Network Open.*

14. Regulate the visiting of others to my child.	○ The nurse does this<br>○ I do this together with the nurse<br>○ I do this independently (without the help of the nurse)<br>○ This is not applicable
15. Participate in the daily rounds with the doctor.	○ The nurse does this<br>○ I do this together with the nurse<br>○ I do this independently (without the help of the nurse)<br>○ This is not applicable
**Domain 3. Acquiring Information**	
16. Did you ask healthcare professionals information on the health of your child?	○ Yes<br>○ No
17. Did you ask the healthcare professionals for information about your child for times when you were not present?	○ Yes<br>○ No
18. Did you talk with another parent about your experiences?	○ Yes<br>○ No
**Domain 4. Parent Advocacy**	
19. I stood up for my child; I told somebody to do something in the care of my child.	○ Yes<br>○ No
20. I stood up for my child; I told somebody NOT to do something in the care of my child; I gave boundaries	○ Yes<br>○ No
21. I gave an explanation on the daily routines of my child to a healthcare professional.	○ Yes<br>○ No
**Domain 5. Time Spent with Infant**	
22. On average, how many hours were you present in the hospital with your child?	Number of hours per day:

© 2022 van Veenendaal NR et al. *JAMA Network Open.*

23. On average, how many hours a day do you have contact with your child?	Number of hours per day:
24. On average, how many hours were you really close with your child?	Number of hours per day:
**Domain 6. Closeness and Comforting the Infant**	
25. Hold/rock/cuddle my child.	○ The nurse does this<br>○ I do this together with the nurse<br>○ I do this independently (without the help of the nurse)<br>○ This is not applicable
26. Comfort my child whenever he/she needs it.	○ The nurse does this<br>○ I do this together with the nurse<br>○ I do this independently (without the help of the nurse)<br>○ This is not applicable
27. Kangaroo care / skin to skin contact.	○ The nurse does this<br>○ I do this together with the nurse<br>○ I do this independently (without the help of the nurse)<br>○ This is not applicable
28. Be together with my child, be close with my child. (intimate time).	○ The nurse does this<br>○ I do this together with the nurse<br>○ I do this independently (without the help of the nurse)<br>○ This is not applicable
29. Be together with my child (be present).	○ The nurse does this<br>○ I do this together with the nurse<br>○ I do this independently (without the help of the nurse)<br>○ This is not applicable
30. Soothe my child during a painful procedure (for instance drawing blood).	○ The nurse does this<br>○ I do this together with the nurse<br>○ I do this independently (without the help of the nurse)<br>○ This is not applicable
31. Recognize my child's signals.	○ The nurse does this<br>○ I do this together with the nurse

© 2022 van Veenendaal NR et al. *JAMA Network Open.*

	○ I do this independently (without the help of the nurse)<br>○ This is not applicable

Based on van Veenendaal et al.[8]

© 2022 van Veenendaal NR et al. *JAMA Network Open.*

eAppendix 2. Supplemental Results

We included fathers of infants who filled out any questionnaires during infant hospital stay in the analyses. Fathers who did not fill out any questionnaires during infant hospital stay were not analyzed, as we did not have any data on their baseline characteristics, such as work status, identification with Dutch cultural background, (see eTable 3, n= 81) and therefore multiple imputation was not deemed fit to perform on this subsample, with too much missingness in characteristics.
Fathers who were non-responders, and thus not included, were not different in their baseline characteristics from responders (eTable 3), yet were still deemed unfit to be included in multiple imputation processes. Similar non-response rates were found in FICare (48.9% vs. 45.7%, p=0.727), the gestational age of their infants was similar (33^{+1} (30^{+6} – 35^{+0}) vs 33^{+3} (31^{+3}- 34^{+6}), p=0.682) and length of stay in the neonatal unit. Thus 81 fathers were not analyzed for this study.
Of those fathers who filled out any questionnaires during hospital stay (n=182), 156 fathers filled out questionnaires at discharge (eTable 4). Thus, 26 fathers filled out questionnaires solely at admission. As we did have data on these fathers, we deemed it fit to include them in analysis and use their data for multiple imputation (see Methods) to generate imputed datasets.

© 2022 van Veenendaal NR et al. *JAMA Network Open.*

eTable 3. Response Rates of Fathers

	Filled out any questionnaires (n= 182)		p-value
	**FICare**	**SNC**	
Fathers <32 (No./Total (%))	42 / 59 (71.2%)	23 / 28 (82.1%)	
Fathers >32 (No./Total (%))	47 / 67 (70.1%)	70 / 109 (64.2%)	
Fathers total (No./Total (%))	89 / 126 (70.6%)	93 / 137 (67.9%)	0.727

*** at discharge and/or admission and/or 3 months of age**

eTable 4. Baseline Characteristics of All Fathers Who Consented to Participate and Did or Did Not Fill Out Any Questionnaires During Infant Hospital Stay

	**Responders (n=182)**	**Non-responders (N=81)**	p-value
FICare (No. (%))	89 (48.9)	37 (45.7)	0.727
Gestational age (weeks, median (IQR))	33^{+1} (30^{+6} – 35^{+0})	33^{+3} (31^{+3}- 34^{+6})	0.682
Inborn (No. (%))	101 (55.5)	48 (59.3)	0.664
Gestational age <32 weeks (No (%))	65 (35.7)	22 (27.2)	0.223
Non-singleton status (No. (%))	28 (15.4)	14 (17.3)	0.837
Paid work (No. (%))	143 (78.6)	Unknown	NA
Identifies with Dutch cultural background (No. (%))	146 (80.2)	Unknown	NA
University degree (No. (%))	149 (81.9)	Unknown	NA
Total length of stay in hospital (birth to discharge, days, median (IQR))	26 (14 – 47)	22 (15 – 34)	0.163
Length of stay in level 2 (admission to discharge, median (IQR))	25 (14 – 37)	20 (14 – 30)	0.125

IQR: interquartile range, No.: number

© 2022 van Veenendaal NR et al. *JAMA Network Open.*

eTable 5. Baseline Characteristics of Included Fathers With or Without Filled Out Questionnaires at Discharge

	**Filled out questionnaires at discharge (n=156)**	**Did not fill out questionnaire at discharge (n=26)**	
FICare (No. (%))	78 (50.0)	11 (42.3)	0.607
Gestational age (weeks, median (IQR))	33+4 (31+0 – 35+1)	32+1 (30+2 – 34+0)	0.048
Inborn (No. (%))	90 (57.7)	11 (42.3)	0.212
Singleton status (No. (%))	131 (84.0)	23 (88.5)	0.769
Paid work (No. (%))	131 (84.0)	12 (46.2)	0.331
Work hours per week (mean (SD))	40.6 (6.6)	37.5 (3.1)	0.0008
Identifies with Dutch cultural background (No. (%))	125 (80.1)	11 (42.3)	0.023
Total length of stay in hospital (birth to discharge, days, median (IQR))	23 (14 – 46)	35.5 (22.8 – 55.5)	0.050
Length of stay in level 2 (admission to discharge, median (IQR))	22 (13 – 36.5)	34.5 (22.8 – 43.5)	0.011
Length of stay in level 3 (admission to discharge, median (IQR))	0 (0 – 6)	2 (0-13.3)	0.197
Gestational age <32 weeks (No. (%))	52 (33.3)	13 (50.0)	0.155
University degree (No. (%))	137 (87.8)	12 (46.2)	0.028
Anxiety and depression score at admission (median (IQR))	6.5 (3 – 11)	5 (3 – 14.5)	0.965
Impaired parent-infant bonding score at admission (median (IQR))	9 (6 – 13)	7.5 (1 – 9.9)	0.204
Self-efficacy score at admission (mean (SD))	59.6 (6.5)	60.9 (6.8)	0.545
Stress total score at admission (mean (SD))	40.5 (16.6)	49.4 (23.0)	0.198
Smoking (No. (%))	14 (9.0)	6 (23.1)	0.0006
Use of drugs (No. (%))	6 (3.8)	0	1.000*
Use of psychotropic drugs (No. (%))	1 (0.6)	0	0.551*
Alcohol use (No. (%))	92 (59.0)	9 (34.6)	0.716

*Fisher exact test, IQR: interquartile range, No.: number, SD: standard deviation

© 2022 van Veenendaal NR et al. *JAMA Network Open.*

eTable 6. Missing Data in Baseline Characteristics of Fathers Included in the Analyses

Characteristic	FICare (n= 89) Missing (No. (%))	SNC (n=93) Missing (No. (%))
Age	2 (2.2)	13 (14.0)
Female	5 (5.6)	7 (7.5)
University degree	7 (7.9)	8 (8.6)
Paid job	7 (7.9)	9 (9.7)
Work hours per week	18 (20.2)	22 (23.7)
Identifies with dutch cultural background	5 (5.6)	7 (7.5)
Stress of pregnancy	5 (5.6)	8 (8.6)
Stress of birth	6 (6.7)	9 (9.7)
Gestational age	0	0
Inborn infant	0	0
Singleton pregnancy	0	0
First child upbringing	6 (6.7)	8 (8.6)
Plan for upbringing Together with partner	6 (6.7)	10 (10.8)
Smoking	11 (12.4)	11 (11.8)
Use of drugs	11 (12.4)	13 (14.0)
Use of psychotropic drugs	2 (2.2)	3 (3.2)
Alcohol use	11 (12.4)	12 (12.9)
Anxiety and depression at admission	54 (60.7)	75 (80.6)
Impaired parent-infant bonding at admission	55 (61.8)	74 (79.6)
Parent self-efficacy at admission	51 (57.3)	73 (78.5)
Stress at admission	51 (57.3)	73 (78.5)

No.: number

Baseline characteristics difference between FICare model and two standard care groups.

After review, we tested for differences between the FICare model and two standard care groups.
For comparison of multiple groups for normally distributed data we used the one-way analyses of variance (ANOVA) and for non-normally distributed data the Kruskall-Wallis test. If differences were significant within the normally distributed data we computed Tukey HSD (Tukey Honest Significant Differences) for performing multiple pairwise-comparison between the means of groups.
For non-parametric tests, we post-hoc performed multiple pairwise U-tests, with a Holm-Bonferroni correction, to test for differences in non-normally distributed data.

Chi-square (χ^2) tests were used to test for differences in binary outcomes. If expected cell-counts were 5 or lower, we calculated differences with the Fisher's exact test. If differences between all groups were significant we performed a pairwise Z-test with Bonferroni correction between proportions of groups.

© 2022 van Veenendaal NR et al. *JAMA Network Open.*

Overall, groups were similar except for gestational age, length of hospital stay or being inborn, but this was not a difference found between the two standard care groups. A difference was present within the standard care group, concerning identifying with a Dutch cultural background(77.9% vs 100%).

eTable 7. Baseline Characteristics Difference Between FICare Model and Two Standard Care Groups

Characteristic	FICare group (n=89)	SNC group 1 (n=75)	SNC group 2 (n=18)	P-value (overall)	P-value (within SNC)
Age (mean (SD))	35.1 (4.8)	36.5 (5.6)	35.5 (4.3)	0.24	
Female (No. (%))	2/84 (2%)	1/68 (1.5%)	0/18	>0.99[a]	
University degree (No. (%))	74/82 (90%)	58/67 (86.6%)	17/18 (94.4%)	0.58	
Paid job (No. (%))	71/82 (87%)	56/66 (84.8%)	16/18 (88.9%)	0.73	
Work hours per week (mean (SD))	39.7 (4.9)	41.2 (8.1)	39.8 (6.1)	0.40	
Identifies with Dutch cultural background (No. (%))	75/84 (89%)	53/68 (77.9%)	18/18 (100%)	0.03[a]	<0.001
Stress of pregnancy (mean (SD), max score 5)	2.1 (1.3)	2.2 (1.4)	2.1 (1.4)	0.75	
Stress of birth (mean (SD), max score 5)	3.2 (1.3)	2.8 (1.2)	2.6 (1.4)	0.07	
Gestational age (median (IQR), range (min-max))	32^{+1} (30^{+1} – 35^{+0}) (24^{+5} – 36^{+6})	34^{+1} (32^{+5} – 35^{+1}) (25^{+4} – 36^{+6})	33^{+1} (31^{+5} – 34^{+1}) (28^{+2} – 35^{+6})	0.02	0.21
Inborn infant (No. (%))	40/89 (45%)	48/75 (64.0%))	13/18 (72.2%)	0.02	0.70
Singleton pregnancy (No. (%))	74/89 (83%)	64/75 (85.3%)	16/18 (88.9%)	0.81	
First child upbringing (No. (%))	61/83 (73%)	45/67 (67.2%)	12/18 (66.7%)	0.66	
Plan for upbringing Together with partner (No. (%))	83/83 (100%)	62/65 (95.3%)	18/18 (100%)	0.09	
Smoking (No. (%))	8/78 (10%)	11/63 (17.5%)	1/18 (5.6%)	0.28	
Use of drugs (No. (%))	4/78 (5%)	2/62 (3.2%)	0/18	0.85[a]	
Alcohol use (No. (%))	47/78 (60%)	43/63 (68.3%)	11/18 (61.1%)	0.60	
Anxiety and depression at admission (median (IQR))	8 (3 – 14) (n=35)	3.5 (1.8 – 5.5) (n=12)	10 (7 – 13) (n=6)	0.08	
Impaired parent-infant bonding at admission (median (IQR))	9 (3 – 12.8) (n= 33)	9 (5 – 12) (n= 12)	10 (9 – 12) (n=7)	0.53	
Parent self-efficacy at admission (mean (SD))	60.4 (6.9)	59.4 (6.5)	58.4 (5.1)	0.71	
Stress at admission (mean (SD))	43.2 (20.1)	44.2 (17.9)	37.4 (11.1)	0.69	
Length stay (days, birth – discharge, median (IQR))	39 (15 - 58)	19 (13.5 – 35)	23 (16 – 38)	0.01	0.59

Data are n/N (%), mean (SD) or median (IQR). Denominators differ because of missing data. [a]: Fisher exact test, IQR: interquartile range, max: maximum, No: number, SD: standard deviation

© 2022 van Veenendaal NR et al. *JAMA Network Open.*

eTable 8. Answers on the PSS-NICU

**Question**	**NA / 0 (n)**	**1 (n)** Not at all stressful	**2 (n)** A little stressful	**3 (n)** Moderately stressful	**4 (n)** Very stressful	**5 (n)** Extremely stressful	**Total answers (n)**
1 The presence of monitors and equipment	12	52	54	22	11	0	151
2 The constant noises of monitors and equipment	24	32	63	20	9	1	149
3 The sudden noises of monitor alarms	25	20	31	49	23	2	150
4 The other sick babies in the room	59	46	12	21	9	2	149
5 The large number of people working in the unit	10	90	23	21	5	0	149
6 Tubes and equipment on or near my baby	19	48	51	20	8	1	147
7 Bruises, cuts or incisions on my baby	74	22	22	16	12	3	149
8 The unusual color of my baby (for example looking pale or yellow jaundiced)	50	23	41	25	6	2	147
9 My baby's unusual or abnormal breathing patterns	35	14	41	31	23	2	146
10 The small size of my baby	13	52	39	33	8	0	145
11 The wrinkled appearance of my baby	64	57	17	6	1	0	145
12 Having a machine (respirator) breathe for my baby	77	15	27	13	11	2	145
13 Seeing needles and tubes put in my baby	32	14	42	25	23	7	143
14 My baby being fed by an intravenous line or tube	16	65	39	16	5	1	142
15 When my baby seemed to be in pain	16	9	42	32	39	4	142
16 When my baby looked sad	16	19	47	36	23	1	142
17 The limp and weak appearance of my baby	50	17	31	23	17	4	142
18 Jerky or restless movements of my baby	19	23	58	30	11	1	142
19 My baby not being able to cry like other babies	89 2	25	19	7	1	1	142
20 Being separated from my baby	24	24	30	34	28	5	145
21 Not feeding my baby myself	48	33	35	19	9	2	146
22 Not being able to care for my baby myself (for example, diapering, bathing)	62	30	25	18	10	1	145
23 Not being able to hold my baby when I want	49	22	30	27	16	2	146
24 Feeling helpless and unable to protect my baby	26	33	36	24	23	4	146

© 2022 van Veenendaal NR et al. *JAMA Network Open.*

from pain and painful procedures							
25 Feeling helpless about how to help my baby during this time	20	33	49	23	19	2	146
26 Not having time alone with my baby	38	37	30	26	12	3	146

n: number

eTable 9. Associations Between Fathers' Participation and Outcomes

**Outcome**	**Beta (95%CI)**	**p-value**	**Adjusted beta (95%CI)***	**p-value**	**Indirect effect**	**95%CI of indirect effect**
Stress	0.254 (-0.140; 0.649)	0.204	0.223 (-0.157; 0.602)	0.248	0.763	-0.627; 2.517
Depression/anxiety	-0.016 (-0.029; -0.0002)**	0.024	-0.015 (-0.028; -0.002)**	0.029	-0.051	-0.133; -0.003
Self-efficacy	0.147 (0.000; 0.294)	0.049	0.133 (-0.022; 0.289)	0.092	0.457	-0.119; 1.357
Impaired father-infant bonding	-0.024 (-0.039; -0.010)**	0.001	-0.024 (-0.040; -0.009)	0.003	-0.082	-0.177; -0.015
Satisfaction with care	0.004 (-0.007; 0.015)	0.479	0.005 (-0.007; 0.017)	0.387	0.018	-0.022; 0.075

Outcomes are from multiple imputed datasets. * adjusted for gestational age, education, cultural background, age, stress at birth, work hours per week, upbringing plan, paternal smoking and alcohol use, **after log transformation, NA: not applicable

© 2022 van Veenendaal NR et al. *JAMA Network Open.*

eReferences

1. Miles MS, Funk S, Carlson J. Parental Stressor Scale: Neonatal Intensive Care Unit. Nurs Res 1993;42(3):148–52.
2. Miles MS, Holditch-Davis D, Schwartz TA, Scher M. Depressive symptoms in mothers of prematurely born infants. J Dev Behav Pediatr 2007;
3. Schappin R, Wijnroks L, Uniken Venema MMAT, Jongmans MJ. Rethinking Stress in Parents of Preterm Infants: A Meta-Analysis. PLoS One 2013;8(2).
4. Brockington IF, Fraser C, Wilson D. The Postpartum Bonding Questionnaire: A validation. Arch Womens Ment Health 2006;9(5):233–42.
5. Brockington IF, Oates J, George S, et al. A screening questionnaire for mother-infant bonding disorders. Arch Womens Ment Health 2001;
6. Latour JM, Duivenvoorden HJ, Hazelzet JA, Van Goudoever JB. Development and validation of a neonatal intensive care parent satisfaction instrument. Pediatr Crit Care Med 2012;13(5):554–9.
7. Barnes CR, Adamson-Macedo EN. Perceived Maternal Parenting Self-Efficacy (PMP S-E) tool: Development and validation with mothers of hospitalized preterm neonates. J Adv Nurs 2007;60(5):550–60.
8. van Veenendaal NR, Auxier J, van der Schoor SRD, et al. Development and psychometric evaluation of the CO-PARTNER tool for collaboration and parent participation in neonatal care. Minor Revis PLOS One
9. Spinhoven P, Ormel J, Sloekers PPA, Kempen GIJM, Speckens AEM, Van Hemert AM. A validation study of the Hospital Anxiety and Depression Scale (HADS) in di?erent groups of Dutch subjects. Psychol Med 1997;
10. Van Bussel JCH, Spitz B, Demyttenaere K. Three self-report questionnaires of the early mother-to-infant bond: Reliability and validity of the Dutch version of the MPAS, PBQ and MIBS. Arch Womens Ment Health 2010;13(5):373–84.
11. Machin D, Campbell MJ, Tan SB, Tan SH. Sample Size Tables for Clinical Studies: Third Edition [Internet]. Wiley-Blackwell; 2009 [cited 2021 Nov 16]. Available from: https://download.e-bookshelf.de/download/0000/6005/91/L-X-0000600591-0001311942.XHTML/index.xhtml
12. Buuren S van. Chapter 6. Imputation in practice [Internet]. In: Flexible imputation of missing data. [cited 2021 Nov 18]. Available from: https://stefvanbuuren.name/fimd/sec-modelform.html

© 2022 van Veenendaal NR et al. *JAMA Network Open.*
